# Dentate granule cells encode auditory decisions after reinforcement learning in rats

**DOI:** 10.1038/s41598-021-93721-8

**Published:** 2021-07-13

**Authors:** Jia Shen, Pan-Tong Yao, Shaoyu Ge, Qiaojie Xiong

**Affiliations:** 1 The Program of Genetics, SUNY Stony Brook, Stony Brook, NY 11794 USA; 2 Department of Neurobiology & Behavior, SUNY at Stony Brook, Stony Brook, NY 11794 USA

**Keywords:** Auditory system, Learning and memory, Neural circuits

## Abstract

Auditory-cued goal-oriented behaviors requires the participation of cortical and subcortical brain areas, but how neural circuits associate sensory-based decisions with goal locations through learning remains poorly understood. The hippocampus is critical for spatial coding, suggesting its possible involvement in transforming sensory inputs to the goal-oriented decisions. Here, we developed an auditory discrimination task in which rats learned to navigate to goal locations based on the frequencies of auditory stimuli. Using in vivo calcium imaging in freely behaving rats over the course of learning, we found that dentate granule cells became more active, spatially tuned, and responsive to task-related variables as learning progressed. Furthermore, only after task learning, the activity of dentate granule cell ensembles represented the navigation path and predicts auditory decisions as early as when rats began to approach the goals. Finally, chemogenetic silencing of dentate gyrus suppressed task learning. Our results demonstrate that dentate granule cells gain task-relevant firing pattern through reinforcement learning and could be a potential link of sensory decisions to spatial navigation.

## Introduction

Successfully navigating to goal/reward locations is critical for the survival of organisms. The transformation of sensory stimuli into motor actions has been extensively studied, with a wide range of brain regions implicated in this process^1–6^. In contrast to the mounting evidence of direct links between sensory representation and motor implementation, however, little is known about how sensory stimulus is associated with goal-oriented actions through reinforcement learning.

The hippocampus, with its tri-synaptic circuit including the dentate gyrus and CA areas, is the key brain substrate for spatial coding^7–10^. Many recent studies have shown that the hippocampus is required in forming spatial memory^11–15^, displaying both stable and dynamic neural representations^16^. Hippocampal neurons acquire responses to auditory conditioned stimuli after fear conditioning^17^ and developed representations for sequential auditory stimuli in non-spatial decision-making behaviors^18^. These findings hint the potential involvement of hippocampus in sensory stimulus guided goal-oriented planning and actions after learning. However, how the hippocampal activity is regulated by task learning and eventually form a representation of the memory is unclear.

Synaptic plasticity was originally found in the dentate gyrus, a subregion of the hippocampus that receives primary afferents^19,20^. The dentate gyrus receives major inputs from the entorhinal cortex, which is considered as the high-level cortical representation^21^. The abundance of the dentate granule cells (DGCs) makes the dentate gyrus a potential neural substrate for encoding new memory^22,23^. Although it has been well accepted that the DGCs exhibit spatial tuned firing pattern^24–26^, its role in goal-oriented behavior learning remains elusive.

To study the role of dentate gyrus activity in learning sensory guided goal-oriented behaviors, we designed an auditory discrimination task in a three-arm chamber with one trigger port and two reward ports at the ends of the side arms. Using in vivo Ca^2+^ imaging, we tracked the activity of DGCs throughout task learning. We found that the firing pattern of DGCs was substantially modulated by learning and formed a stable representation of the auditory-cued decisions and stronger response to task-related events. Importantly, the DGC population showed distinct activity patterns to represent different decisions only after learning and only when engaged in the task. Furthermore, DGCs activity is required for acquiring the task. Our results suggest that the dynamics of dentate gyrus may convey sensory decisions to navigation during reinforcement learning.

## Results

### Auditory task learning enhances DGC activity and spatial tuning

**Figure 1:**
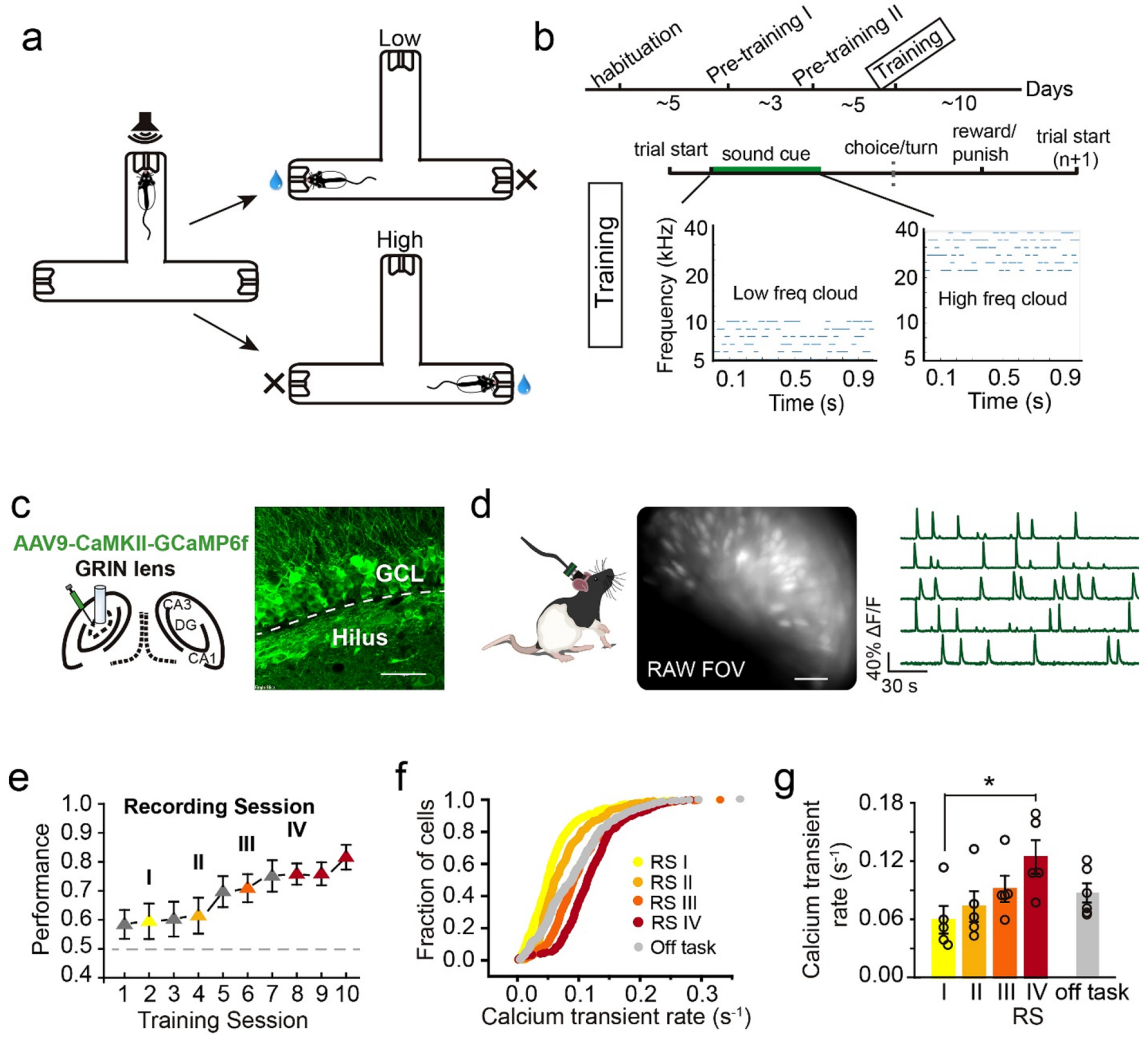
DGC activity intensified during auditory task learning. (**a**,**b**) Schematics of an auditory-cued decision task and the training timeline. Low or high frequency tone clouds were associated with a reward in the left or right port, respectively. (**c**) Left, schematic of AAV-GCaMP6f injection and lens implantation in the DGC layer. Right, expression of GCaMP6f in DGCs (scale bar: 50 µm, GCL: granule cell layer). (**d**) Cell detection with the miniature microscope (raw view) in a freely moving rat. Example Ca^2+^ signal traces from the signal processing algorithm CNMF-e. (**e**) Task learning progress on task performance. The Ca^2+^ imaging sessions were labeled yellow, light orange, dark orange and red, indicating learning progress (gray: sessions without Ca^2+^ recording). (**f**) Ca^2+^ transient rate in all recording sessions and an off-task session (RS I: 529 cells, RS II: 458 cells, RS III: 515 cells, RS IV: 359 cells, Off task: 412 cells). (**g**) Quantification of Ca^2+^ transient rate in each recording session (RS) and an off-task sessions (n = 5 rats, mean ± SEM, Kruskal–Wallis test, *P < 0.05).

To examine the role of the hippocampus in associating a sensory-based decision to a goal location during spatial navigation, we designed an auditory-cued goal-oriented task in which we monitored and manipulated the activity of DGCs. In this task, a rat learns to self-initiate a trial by poking its nose into the center port, which triggers an auditory stimulus consisting of a cloud of tones with low (5–10 kHz) or high (20–40 kHz) frequencies (Fig. 1a,b). Based on the frequencies of tones, a rat navigates to one of the side ports (e.g., low frequencies—left side vs. high frequencies—right side) for a water reward. Auditory stimuli stop once the rat withdraws from the center port. It takes 2–4 weeks for a rat to learn the task. Well-trained rats had an average reaction time (from sound onset to withdraw from the center port) of ~ 0.181 s in correct trials and ~ 0.142 s in error trials.

To investigate how DGCs participate in the auditory-cued goal-oriented task, we used in vivo Ca^2+^ imaging^27–29^ to track the activity of about 400 (RSI: 530 cells, RS II: 459 cells, RS III: 360 cell, and RS IV: 413 cells) DGCs from 5 rats during learning. We injected AAV expressing a Ca^2+^ indicator, GCaMP6f^30^, into the dentate gyrus followed by GRIN lens implantation and miniaturized microscope installation (Fig. 1c). We recorded Ca^2+^ activity at least ~ 4 weeks after virus injection to ensure sufficient and stable GCaMP6f expression (Fig. 1c,d, Supplementary video). We then downsampled the raw recording videos and performed motion correction with Mosaic software (version 1.2; Inscopix Inc. detail in “Methods”). Ca^2+^ signal traces were extracted using CNMF-e^31,32^.

After viral injection and lens implantation, we habituated rats to the task chamber daily for 5 consecutive days (30 min per day) before training to circumvent any changes induced by exploring a new environment as previously reported^16,33^. Rats were then trained in the auditory-cued decision task for one session per day. After 10 training sessions in the Training Phase (Fig. 1b and “Methods”), rats performed with ~ 81% accuracy (Fig. 1e and Supplementary Fig. 1a). To examine changes in DGC activity during learning, we performed Ca^2+^ imaging on DGCs every other session as well as two additional sessions after well trained (Fig. 1e). There were no differences in behavioral reaction time across sessions and a slight trend of increased running speed in late training sessions (Supplementary Fig. 1b,c). We then calculated the Ca^2+^ transient rate for all cells detected in the recording sessions. At the beginning of learning, the DGC Ca^2+^ transient rate was 0.06 s^−1^ (± 0.014 s^−1^), comparable to that analyzed with electrophysiological and two-photon imaging approaches^24,25^, and it increased as learning progressed to 0.124 s^−1^ (± 0.017 s^−1^, Fig. 1f,g and supplementary Fig. 1d). This increase was unlikely due to increased viral expression in the later training sessions, as the Ca^2+^ transient rate was similar between an off-task recording session after learning (i.e., well-trained rats freely exploring the same chamber with randomly played sounds) and recording session II (off-task: 0.087 ± 0.01 s^−1^, RS II: 0.073 ± 0.016 s^−1^, Kruskal–Wallis test, n.s., *P* = 0.715, Fig. 1f,g).

**Figure 2:**
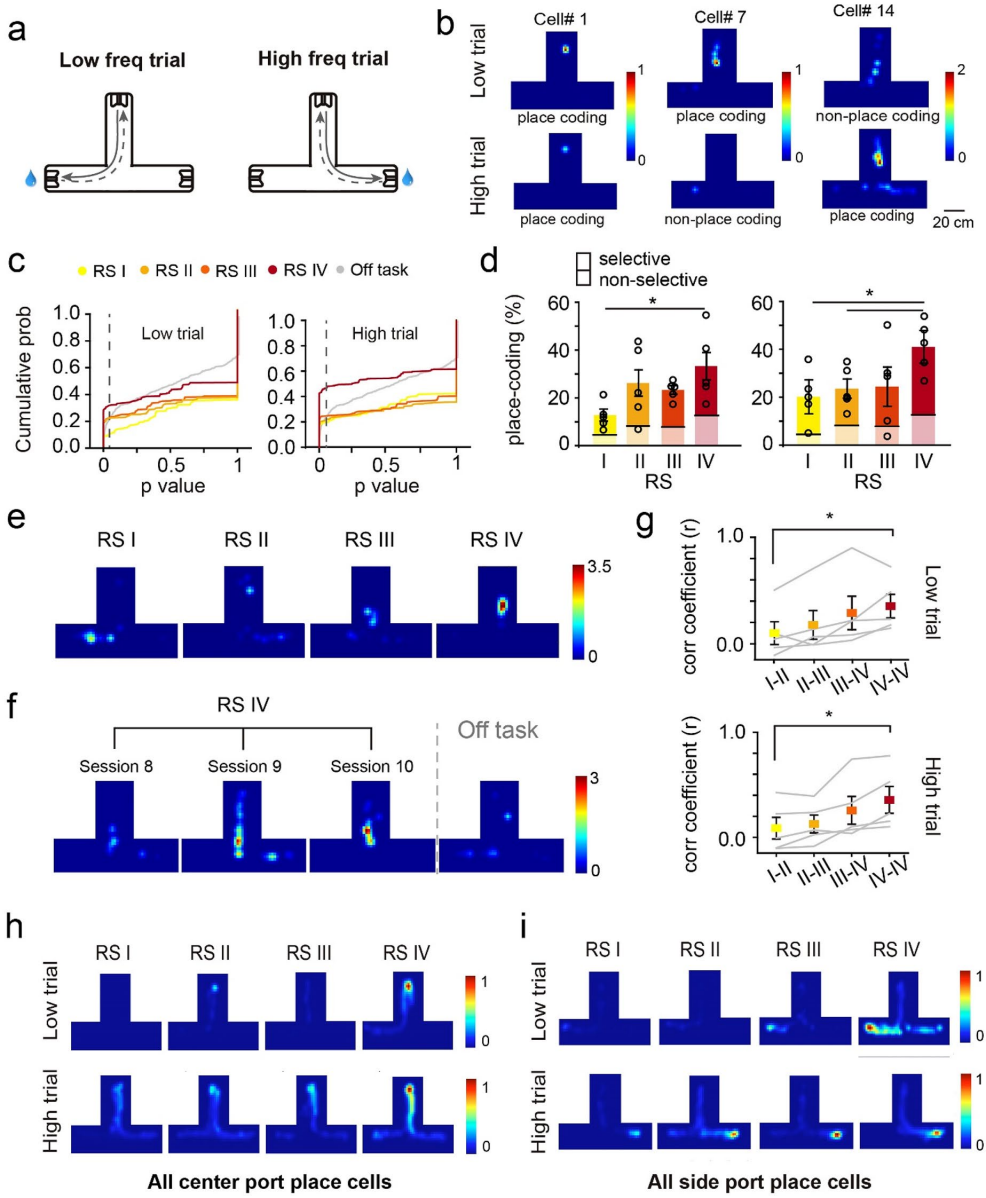
Dynamic place-coding in the dentate gyrus during the auditory task learning. (**a**) Schematic of low and high trial routes. (**b**) example place-coding cells. (**c**) Cumulative probability of the *P* values in spatial information analysis in each learning RS and off-task session in low (left) and high (right) trials (dashed line indicates the place cell threshold *P* value, 0.05). (**d**) Quantification of the ratio of place-coding cells in low (left) and high (right) trials (solid color: trial type-selective cells, transparent color: non-trial type-selective cells, n = 5 rats, mean ± SEM, Kruskal–Wallis test, *P < 0.05). (**e**) Example of DGC remapping during learning. (**f**) Example of DGC showing stable place fields in the three sessions of RS IV and remapping in the following off-task session. (**g**) Correlation coefficients of Ca^2+^ transient events between two recording sessions and different sessions in RS IV in low (top) and high (bottom) trials (n = 5 rats, mean ± SEM, Kruskal–Wallis test, *P < 0.05). (**h**) Ca^2+^ transient rate overlay heatmap of all the place cells that have the highest firing rate near the center port in low or high trials in each RS (color bar, normalized Ca^2+^ transient rate to that in RS IV sessions for comparison). (**i**) Ca^2+^ transient rate overlay heatmap of all the place cells that have highest firing rate near the side port in low or high trials in each RS (color bar, normalized Ca^2+^ transient rate to that in RS IV for comparison).

DGCs are either quiescent or exhibit a single place field in a given environment^16,25,26,34^. We thus characterized the place-coding profile of detected active DGCs during learning. To determine whether DGC spatial representation was modulated by task learning, we analyzed place-coding DGCs using spatial information (see “Methods”) separately in low and high sound frequency trials (Fig. 2a). We found that 12.8% and 20.2% of detected active cells (92 ± 16 cells) were place-coding in low- and high-frequency trials in recording session I, respectively (Fig. 2a–c). However, regardless of whether individual neurons were place-coding in both trial types or specifically in one type, the percentage of place-coding cells substantially increased in later learning sessions to 37.1% and 43.4% (out of 78 ± 16 cells), respectively (Fig. 2c,d and Supplementary Fig. 1e).

We further analyzed the place-coding profile of individual DGCs during learning by registering cells across recording sessions (Supplementary Fig. 2a–d). We identified about half of the detected cells in all four recording sessions using Cell Reg^35^. Among these registered cells, the place fields of individual DGCs underwent dramatic remapping over the course of learning and became stable upon reaching recording session IV (198 cells, Fig. 2e–g and Supplementary Fig. 2f). Interestingly, the stable representation in recording session IV was present only in task but not in off-task recording sessions (correlation coefficient between RS IV and off-task is 0.124 ± 0.024; 0.353 ± 0.11 and 0.356 ± 0.128 between two sessions in RS IV in low and high trials, respectively, Fig. 2f,g). The average correlation coefficient of the spatially binned Δf/f was also higher in later recording sessions (Fig. 2g). In recording session IV, more individual DGCs displayed stable place fields across multiple sessions (RS I: 28% ± 11.6%; RS IV: 62% ± 8%, Supplementary Fig. 2f). Consistently, the average relative distance of place fields between sessions was significantly larger in early learning recording sessions than within recording sessions IV (Supplementary Fig. 2f). Importantly, more place cells were aggregated near the center and side ports (Fig. 2h–l and Supplementary Fig. 2g,h) where the rats received sound cues and reward. Our findings suggest that task learning increased the spatial tuning of DGCs, stabilized their place fields, and specifically enhanced their in task-related locations.

### Emergence of event-specific DGCs activities during auditory task learning

**Figure 3:**
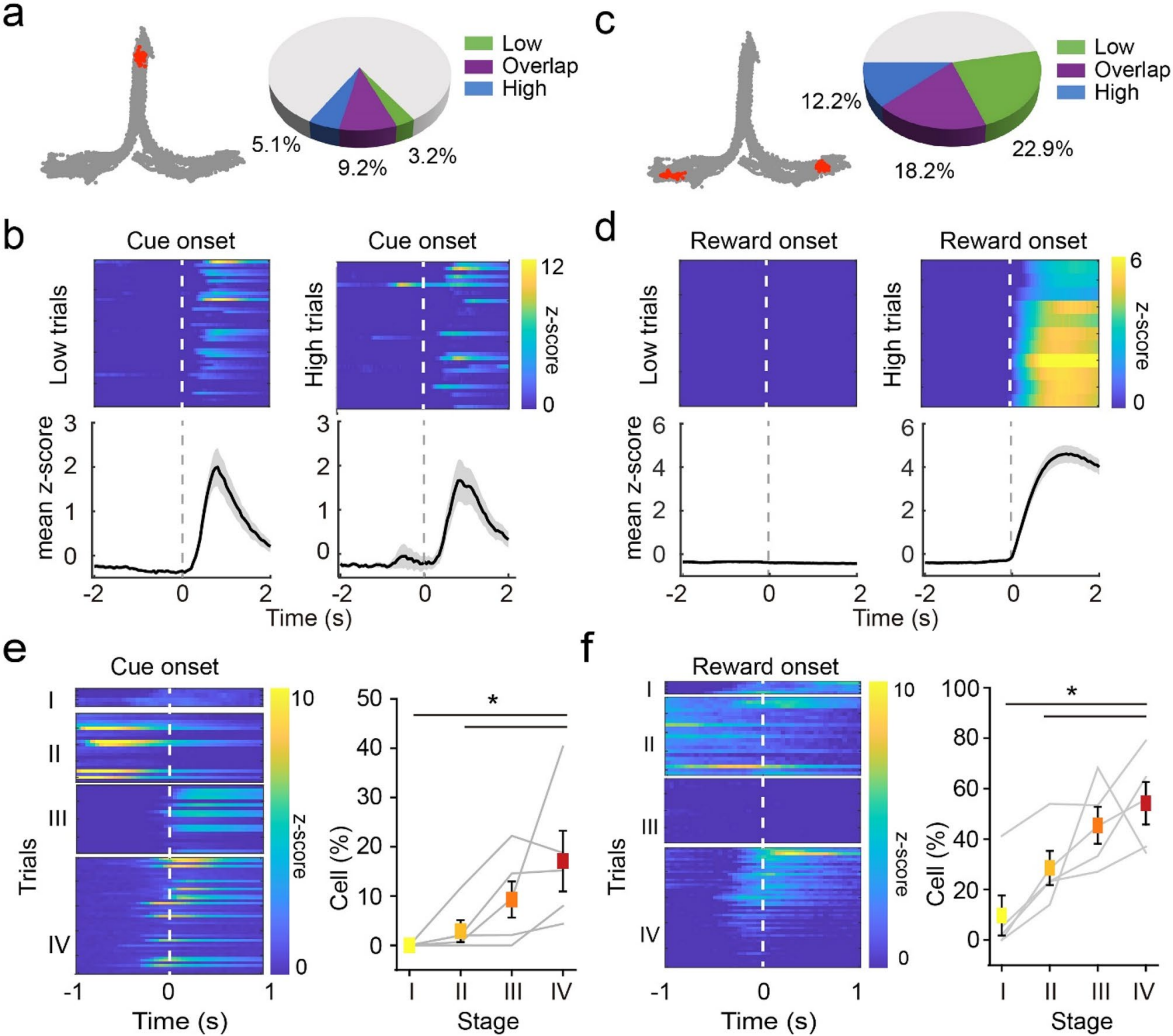
DGC encodes task-related variables in the auditory task. (**a**) Left, example behavioral trajectories (gray) and location at cue onset (red). Right, composition of low and high trial-responding and overlapping cells. (**b**) Example cell that presented higher Ca^2+^ signal post-cue onset in both low (left) and high (right) trial. Top, z-scored Ca^2+^ intensity aligned to cue onset in each trial. Bottom, trial-averaged z-score. (**c**) Left, example behavioral trajectories (gray) and location at reward onset (red). Right, composition of low and high trial-responding and overlapping cells. (**d**) Example cell that presented higher Ca^2+^ signal post-reward onset in high but not in low trials. Top, z-scored Ca^2+^ intensity aligned to reward onset in each trial. Bottom, trial-averaged z-score. (**e**) The emergence of cue-onset cells over the course of learning. Left, example cell that was not cue-responding in early learning process but emerged to be active in later RS. Right, percentage of cue-responding cells in each RS (n = 5 rats, mean ± SEM, Kruskal–Wallis test, *P < 0.05). (**f**) The percentage of reward-onset cells increased across learning. Left, example cell that was not responding to reward or anticipating reward but emerged to be active near the reward location in later RS. Right, percentage of responding cells in each RS (n = 5 rats, mean ± SEM, Kruskal–Wallis test, *P < 0.05).

As the DGC firing profile was substantially modulated over the course of learning, we postulated that the learning-shaped DGC activity encodes task-relevant information. To test this hypothesis, we aligned individual DGC activity to cue-onset (Fig. 3a,b) and reward-onset (Fig. 3c,d) time points. We found that 17.49 ± 6.28% and 54.18 ± 8.4% of the detected cells showed significantly higher Ca^2+^ fluorescent signal after cue-onset and reward-onset, respectively (Fig. 3a,c, also see “Methods”).

Notably, around half (49.96 ± 10.74%) of cue-onset cells preferentially responded after either low or high frequency sound cue that triggered by center-port nose poking (Fig. 3a), whereas the other half responded indistinguishably between two trial types (Fig. 3a,b). To test whether the non-selective cue responding cells are simply place coding cells, we analyzed the DGC activity in early-withdrawal trials whereby the rats didn’t poke the center port long enough to trigger the sound cue. We found that over 70% of the cue responding cells showed little response after center port poking without receiving sound cues (Supplementary Fig. 3a,b,e and see “Methods”). We further analyzed the DGC activity based on decision outcomes (i.e. correct or error choice). Around 69.8% of reward-onset cells preferentially responded in either low or high trials, whereas 30.2% of reward-onset cells responded in both trial types although the two reward ports were in different locations (Fig. 3c,d and Supplementary Fig. 3c,d). Similarly, the majority of the reward-onset responding cells were not activated in error trials when the reward was absent (Supplementary Fig. 3c,d,f). This task event-related DGC activity emerged as learning progressed, with ~ 17.5% and ~ 44.5% increases in cue-onset and reward-onset cells from session I to IV, respectively (Fig. 3e,f). These task-associated cells were not necessarily pre-defined place-coding cells (Supplementary Fig. 3g). These findings suggest that DGCs form an activity representation during learning that may play an essential role in auditory-cued decision-making. Interestingly, ~ 80% of cue-onset cells were selectively active in correct trials but not in error trials (Supplementary Fig. 3h), indicating that the DCG activity plays a role in decision-making.

### DGC ensembles convey decision-related information in the auditory task

**Figure 4:**
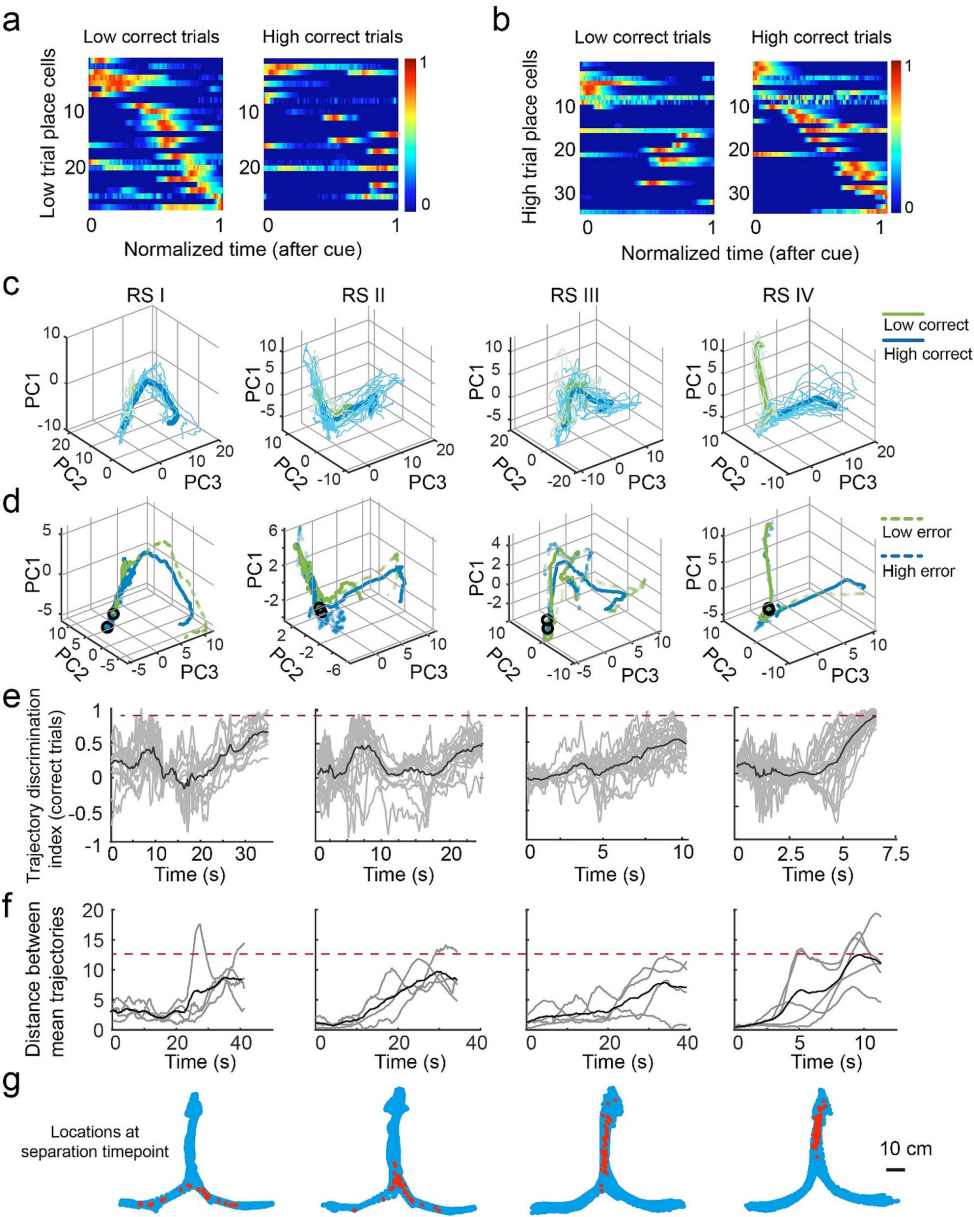
DGC ensembles convey information related to auditory decisions. (**a**,**b**) Sequential activity of place-coding cells in low and high trials and distinct sequences of place-coding DGCs in different trial types. (**c**) Example neural trajectories in low (green) and high (blue) correct trials in a 3-D space. Thin lines indicate individual trials, and thick lines indicate the mean trajectory. (**d**) Solid lines indicate the mean trajectory in correct trials, and dashed lines indicate the mean trajectory in error trials (e.g., the green dashed line corresponds to low trials in which the rat made an error). (**e**) Neural trajectory discrimination index in correct trials (gray, single trial index; black, averaged index), which indicates the similarity of a single trial trajectory to its own (index = 1) or opposite (index = − 1) trial type averaged value. (**f**) Distance between trajectories of two trial types (gray, single rat distance; black, averaged rat distance). (**g**) Locations of rats when the neural trajectories in two trial types separated.

The learning-induced firing patterns of DGCs suggest that these cells carry information other than location, such as information related to sensory perception, decisions, and/or the reward. Therefore, we investigated whether the spatial representations of DGCs represent rats’ decisions by determining whether the information carried by DGCs is a combination of clustered cells (i.e., cue-onset and reward-onset cells) or high-dimensional coding. We first computed Pearson’s correlations between non-averaged fluorescence intensity from each pair of cells in correct trials of each trial type. Most pairs (73.5% for low and 66% for high trials) showed small correlation coefficients (r < 0.2). This suggests that cells were activated at different time points within trials and thus may fire in a sequential pattern throughout the trial. Indeed, the z-scored, trial-averaged Δf/f of place-coding DGCs had peak signals at different time points, and the cross-validated sequential activity covered the entire trial from cue onset to reward onset (Fig. 4a,b). Place-coding DGCs in one trial type did not exhibit the sequential pattern in the other trial type (Fig. 4a,b and Supplementary Fig. 4a–f), suggesting that this sequential pattern is not simply encoding timing and distinct DGC ensembles participate in different auditory-cued decisions. Similarly, we found trial type specific sequential DGC activity over the rat’s physical linearized location in the maze (Supplementary Fig. 4c,d). This sequential firing pattern of DGC ensembles throughout the entire trial suggests a high-dimensional coding mechanism.

To further understand how DGCs encode auditory-cued decisions as a population, we computed the neural trajectory^36^ for each trial type. Principal component analysis (PCA) was performed to reduce dimensionality, and the three components explaining the most variance were used to visualize the neural trajectory from the cue onset to the trial end in three-dimensional space. We found that averaged neural trajectories separated the two choices in correct trials in recording session IV and individual trials were more similar to the mean of its own trial type than that of the opposite choice (Fig. 4c–g). Interestingly, neural trajectories in error trials became strongly discriminative toward the opposite choice in recording session IV (Fig. 4d and Supplementary Fig. 4g). By contrast, the neural trajectories in early recording sessions were not as well divergent in space (Fig. 4c,d). These trajectories only showed modest discrimination indices (Fig. 4e) and smaller distances (Fig. 4f) between two decision trajectories. These results suggested that the DGC represents rats’ spatial decisions in a cell population level and this dynamics only occurred after learning.

### Auditory decisions can be decoded from the activity of DGC ensembles

**Figure 5:**
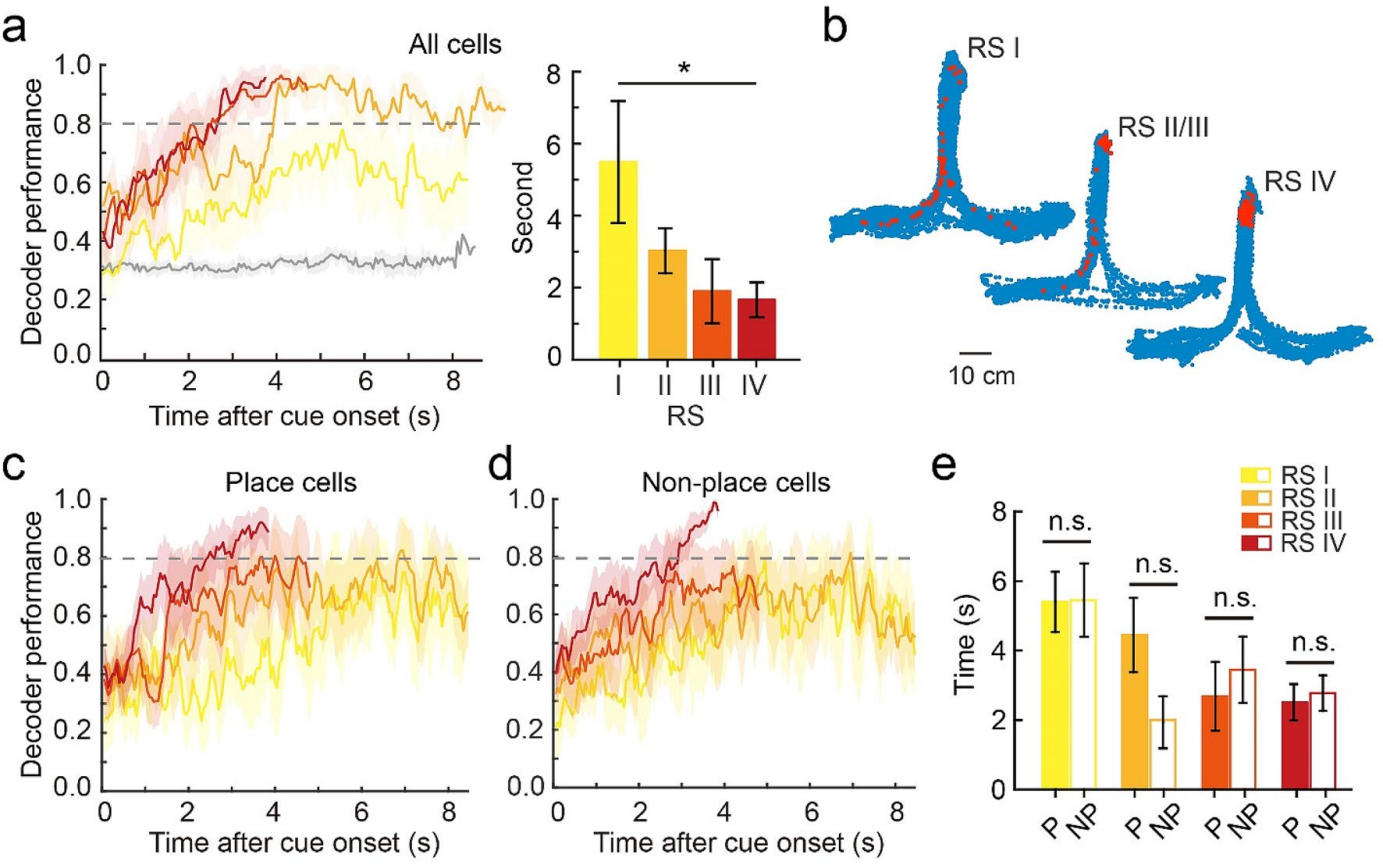
Activity of DGC ensembles represents auditory decisions. (**a**) Left, decoder performance in each frame after cue onset in each learning RS showing the maximum frame number per trial for each rat (gray line indicates the shuffled data in RS I). Right, quantification of frames needed to reach 80% decoder performance after cue onset (n = 5, Wilcoxon signed-rank test, mean ± SEM, *P < 0.05). (**b**) Trajectory with decoder location in each RS (blue: rat’s location across the entire session, red: rat’s location at the frame with 80% decoder performance). (**d**,**e**) Place-coding and non-place-coding decoder performance. (**e**) Quantification of frames needed to reach 80% decoder performance after cue onset using only place-coding (P) or non-place-coding (NP) cells (n = 5 rats, mean ± SEM, Wilcoxon signed-rank test, n.s., *P* > 0.05).

Finally, we aimed to decode rats’ decisions using DGC ensemble activity. We employed a Support Vector Machine (SVM) classifier to decode decisions using the non-averaged Ca^2+^ traces (20 Hz, non-spatially binned) from all cells in correct trials. In recording session IV, decoder performance reached 80% accuracy as early as 1.75 s after the start of trials (Fig. 5a). However, the SVM decoder poorly predicted decisions in early recording sessions. To rule out the potential influence of trial number on decoder performance, we balanced the number of trials in early and late recording sessions and still found better performance in recording session IV (Supplementary Fig. 5a–d). We also tested whether groups of coactivated DGCs or isolated neurons encode auditory decisions by breaking the temporal correlation between DGCs. Each neuron’s Ca^2+^ trace in RS IV was shifted by − 2, − 1, 0, 1, or 2 s as the input data for the decoder. We found that the temporally shifted data reached 80% decoder performance slightly later than the real data (Supplementary Fig. 5e).

The finding that DGC ensembles decode trial types suggests that DGCs link auditory decisions to spatial navigation. However, it could also be argued that some DGCs display differential activity in left vs. right reward arms of the chamber due to the physical locations of rats (i.e. place cells). To test this possibility, we examined rats’ physical locations at time points at which decoder performance reached 80% accuracy. In early recording sessions, rats turned to the side arms in 42% of trials in which the average time decoder performance was above 80%, whereas in recording session IV, rats were located in proximity to the center port (Fig. 5b). This finding suggests that DGC ensembles actively encode the rats’ decisions rather than passively reflecting their physical locations.

We further examined whether only place-coding DGCs contribute to decoder performance. We divided DGCs into place-coding or non-place-coding populations (based on the criteria shown in Fig. 2b) and utilized the same SVM classifier to decode auditory decisions. Interestingly, the two groups of cells equally contributed to the decoding of auditory decisions (Fig. 5c–e). Taken together, these results suggest that DGC ensemble activity conveys information related to auditory-cued decisions and develops during learning.

### Silencing DGCs impairs auditory task learning

**Figure 6:**
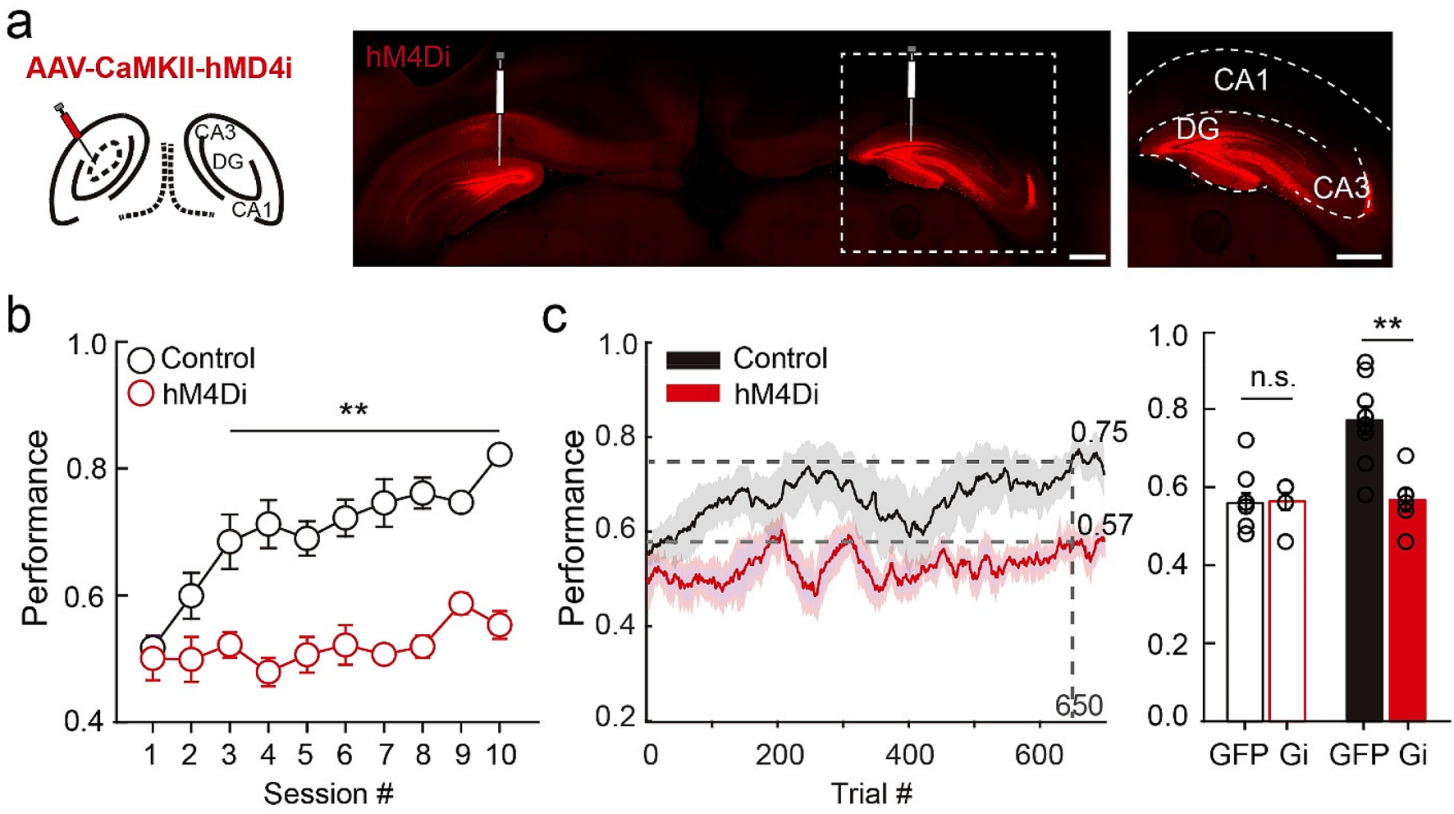
DGC activity is required for learning the auditory task. (**a**) Confocal images of the hippocampus with hm4Di-mCherry expression in the dentate gyrus (scale bar: 1 mm). Right, enlarged image from the white dashed box (scale bar: 1 mm). (**b**) Session performance. Control rats reached ~ 75% accuracy within 10 sessions, whereas hM4Di-injected rats performed at chance level (n = 10 rats for control and n = 6 rats for hM4Di, mean ± SEM, Kruskal–Wallis test, **P < 0.01). (**c**) Trial performance (in 100-trial sliding window). Control rats reached ~ 75% performance by the 650th trial, whereas the hM4Di-injected rats performed at chance level. Right, average performance in the first 100-trial window and the 650th window (n = 10 rats for control and n = 6 rats for hM4Di, mean ± SEM, Kruskal–Wallis test, n.s., *P* > 0.05, **P < 0.01).

We next examined whether DGCs are required for learning this task by inhibiting their activity during training. Naïve rats were bilaterally injected with adeno-associated virus (AAV) expressing DREADD AAV-CaMKII-hM4Di-mCherry^37,38^ or AAV-CaMKII-GFP (control) into the dorsal dentate gyrus (Fig. 6a). Two to three weeks after surgery, when the AAV-delivered transgenes are expressed^29^, we started training with one session per day. Rats were administered CNO (5 mg/kg, i.p.) 30 min before each session and were trained during the ~ 1-h period during which CNO is effective^37,38^. Whereas control GFP-injected rats achieved ~ 75% accuracy after 5.7 ± 2.4 training sessions in the Training Phase, the performance of hM4Di-injected rats remained at chance levels after 10 training sessions (Fig. 6b). Reaction time did not differ between control and hM4Di rats (hM4Di rat: ~ 0.208 s in correct trials and ~ 0.174 s in error trials, Supplementary Fig. 6a,b), suggesting that hM4Di expression in DGCs did not impact motor function or strategy. As rats required less time per trial after mastering the task (Supplementary Fig. 6c,d), hM4Di rats completed fewer trials in later training sessions. To rule out the possibility that the number of trials per session affected learning progress, we analyzed performance using a 100-trial sliding window. Consistent with the analysis by session, control rats reached 77.1 ± 10.7% accuracy after ~ 650 trials, whereas the performance of hM4Di rats was 56.7 ± 7.1% after the same number of trials. Initial performance during the first 100 trials, however, was comparable (Fig. 6c). These results suggest that DGCs participate in learning the auditory-cued goal-oriented task, which requires the utilization of spatial signals.

## Discussion

In this study we investigated whether and how DGCs participate in the auditory-cued goal-oriented behaviors. We found that reinforcement learning modulated the firing rate and spatial tuning of individual DGCs. During the course of learning, more DGCs were responsive to task-related events, such as auditory stimuli and reward. Only after the learning, the activity of DGC ensembles represented navigation path and predicted choices. Furthermore, the DGCs activity is required for the task learning. Our findings reveal that DGCs associate spatial information with sensory decisions during goal-oriented behavior through reinforcement learning.

### DGC activity remaps during auditory-cued decision task learning

We found that after learning an auditory-cued decision task, DGC activity was increased and strongly correlated with specific locations. During the learning process, DGCs exhibited highly dynamic remapping (Fig. 2) and eventually constructed a representation of the behavioral task in a spatial-dependent fashion (Figs. 3, 4). The cognitive maps of CA1 pyramidal cells^12–15,39^ are dynamic neural representations of the animal’s internal perspective of the external space. Our findings suggest that DGCs form similar maps in the dentate gyrus after auditory task learning. We performed 5 days of habituation in the decision task apparatus to exclude the neural dynamics from learning the context, however, Kim et al.^40^ recently showed that the slow emergence of dentate spatial maps plateaus after a week. This could potentially explain the neural activity pattern in “off task” assemblies to RS II or III more than RS I. We point out that applying SVM to DGC Ca^2+^ signals only decoded auditory-cued decisions in late but not early learning sessions (Fig. 5), suggesting that this cognitive map does not solely reflect rats’ physical trajectories. Training animals in a spatial alternation task might produce similar neural dynamics in the hippocampus that discriminates left and right routes^33^. How do DGCs contribute to sensory evidence accumulation during learning, and which neural circuits enable this association? Further studies are required to answer these questions. We also found that non-place-coding DGCs contributed equally well as place-coding DGCs in SVM decoder performance (Fig. 5c–e) and that task-associated cells (Fig. 3) were not necessarily place-coding. These observations suggested that those event encoding, non-place cells might fire at multiple locations in the maze or preferentially fire when the rat was stopping, therefore are no long typical place cells. These results implies that the internal network state of DGCs is crucial for encoding planning and decisions, which might or might not related to space.

### DGCs carry reward signals

It was suggested that the CA1 allocates a small dedicated population of neurons to encode reward that is independent of locations^41^. Interestingly, we also found a subset of DGCs preferentially active around reward onset (Fig. 3c,d,f). Whether and how this signal is involved in task learning and performance remains elusive. DGCs receive both dopaminergic and non-dopaminergic projections from the ventral tegmental area^42–44^, which hints at a reward expectation and/or feedback component of the modulation of DGC activity during learning. Further studies could determine which reward-related information (e.g., expectation, valence) is carried by these DGCs and how they contribute to learning or the maintenance of performance.

### Circuits linking auditory-cued decision-making to the dentate gyrus

We demonstrate that DGCs encoded auditory-cued decisions after learning, but how might these decisions be relayed to DGCs? The auditory striatum receives projections from the primary auditory cortex and medial geniculate body and conveys learned associations between sound stimuli and reward port locations^3^. However, there is no direct connection between these auditory regions (i.e., medial geniculate body, primary auditory cortex, auditory striatum) and the dentate gyrus. Determining whether auditory-cued decisions are delivered to DGCs through motor planning/initiation regions (e.g., motor cortex, basal ganglia) or higher-order cognitive cortices (e.g., parietal cortex, entorhinal cortex) would be of great interest. The temporal shifting decoder analysis (Supplementary Fig. 5e) only showed a trend (but not statistically significant) of decreased performance using non-correlated DGC activities. This suggested that although selected neurons were coactivated in each auditory decision whereas only a subset of them is sufficient to send discriminative information to the downstream brain region.

### Spatial representation is required for auditory-cued goal-oriented behaviors

In daily life, decisions based on sensory stimuli are likely associated with spatial representations and direct navigation to goal locations. In the present study, we found that hippocampal DGCs carrying spatial representations are required for learning to make auditory-cued decisions (Fig. 6), suggesting that the dentate gyrus may be a locus linking sensory-based decisions to spatial navigation. We note that the virus expressing CaMKII-hM4Di for neuronal silencing targeted both DGCs and mossy cells, both of which encode spatial information^25,45^. Although in this study we focused on the role of DGCs in establishing representations of auditory-cued decisions during learning, future studies could separately manipulate DGCs and mossy cells.

Altogether, our findings suggest that spatial information in the dentate gyrus plays an essential role in sensory-based goal-oriented learning and behaviors.

## Methods

### Rats and virus

Animal procedures were approved by the Stony Brook University Animal Care and Use Committee and carried out in accordance with National Institutes of Health standards. Viral injections were conducted using 4-week-old male and female Long Evans rats (Charles River Laboratories). Rats were housed on a 12-h light/dark cycle, and all behavioral experiments were performed during the dark phase. Rats were provided ad libitum access to food and water. AAV9- CaMKII-GCaMP6f was purchased from the University of Pennsylvania Vector Core, and AAV8-CaMKII-hM4Di-mCherry and CaMKII-GFP were purchased from Addgene.

### In vivo Ca^2+^ imaging in freely moving rats

AAV9-CaMKII-GCaMP6f virus was stereotactically injected into the dorsal dentate gyrus (AP: − 4.0 mm, ML: 2.6 mm, DV: 3.5 mm) as previously described^46^ when rats were 4-week-old for better virus spread. After ~ 3 weeks of viral expression (i.e., when rats were 7–8 weeks old), a lens probe (outer diameter = 1.0 mm, length = 9.0 mm, numerical aperture = 0.5) was implanted ~ 200 µm above the DGC layer. Another ~ 3 weeks later, a microscope baseplate was fixed to an optimized focal plane on top of the lens probe. All Ca^2+^ imaging videos were recorded using a miniature microscope and the nVista data acquisition system (Inscopix, Palo Alto, CA) at a frame rate of 20 Hz. LED power was usually < 60%, and the length of recording sessions was ~ 20 min to avoid photobleaching. Miniature microscopes were attached to rats immediately before recording and removed immediately afterward. The focal plane was kept the same throughout the entire recording period for successful longitudinal cell registration.

### Behavioral task

Rats were placed in the center of a three-arm chamber (100 cm × 70 cm) daily for 30 min for free exploration for 5 consecutive days before training. The water bottle was then removed from their home cages, and 10–15 g hydrogel (ClearH2O, Portland, ME) was provided per day for water deprivation. Training and testing in the auditory-cued decision task consisted of three phases, with one session per day lasting ~ 1 h. In the Pre-training Phase I, rats were trained to poke their nose into the center port to trigger the start of a trial, after which a water reward was delivered to a side port. During this phase, water was immediately delivered to the correct side port after poking the center port. In the second Pre-training Phase, poking at the correct side port was necessary to trigger water delivery; however, rats had 30 s to poke the correct port, and poking the wrong port during the trial had no consequence. In the Training Phase, a poke in the center port led to a fixed 0.05 s delay followed by a tone cloud played in a low (5–10 kHz) or high (20–40 kHz) frequency range indicating the reward location. Each tone cloud was 1 s maximum duration but stopped when rats withdrew from the center port. The purpose of the first two phases was to allow the animal to learn the layout of the task. All behavioral and DGC firing pattern results were from the Training Phase. The behavioral task was controlled by a MATLAB-based system, Bpod (Sanworks), which randomly generated the trial type and calibrated tone frequency and power. Rat locomotion was tracked by EthoVision XT 13 (Noldus Information Technology Inc.). Ca^2+^ imaging was acquired using nVista 2.0 (Inscopix) installed on another computer to avoid potential hardware or software conflicts. The nVista recording was triggered by external hardware (EthoVision) to synchronize the Ca^2+^ signal and locomotion data. The Bpod system also sent BNC pulses to nVista as timestamps for the cue and reward onsets. For the dentate gyrus inhibition experiment (Fig. 6), CNO was injected (5 mg/kg, i.p.) 30 min before the start of sessions.

### Data processing and analysis

Ca^2+^ imaging recordings within individual rats with the same field of view were concatenated and motion-corrected (Mosaic, Inscopix). Active cells in each video were detected by the MATLAB-based algorithm CNMF-e^31,32^. Individual cells detected by the algorithm were screened manually afterwards. Only cells with clearly identified signals and contour were included. Denoised fluorescence signal traces were used to detect Ca^2+^ transient events. Longitudinal cell registration was performed in CellReg^35^ with the spatial components extracted from CNMF-e. All data processing and analysis was performed in MATLAB.

### Place-coding cells and behavior epoch-responding analysis

Place-coding cells were identified based on each cell’s spatial information^47^. Briefly, the three-arm chamber was divided into 19 spatial bins, and the Ca^2+^ transient rate in each spatial bin was calculated. The spatial information of each cell (I_N_) was computed using the equation ${I}_{N}={\sum}_{i=1}^{N}{\lambda}_{i}ln\frac{{\lambda}_{i}}{\lambda}{\rho}_{i}$, where N is the total number of spatial bins, λ is the overall firing rate, and λ_i_ and ${\rho}_{i}$ are the Ca^2+^ transient rate and time spent in the bin, respectively^24^. I_N_ was compared to 10,000 times of shuffled spatial information (I_S_) with randomly generated transient event times. *P*-values were calculated as 1 − (I_N_ > I_S_)%, and cells with *P* < 0.05 were considered place-coding cells. In the behavior epoch-aligned analysis (Fig. 3), fluorescence intensity from the 10 frames before cue or reward onset from all trials was taken as a baseline measure. The difference between the baseline mean and the mean of up to 10 frames after each behavioral epoch onset was calculated. The pseudo-mean fluorescence difference was computed 10,000 times and compared to the real mean difference. The P value is defined by the probability of the real mean difference being smaller than the pseudo-mean difference. The best P values from all cells were then adjusted by false discovery rate (FDR) correction at 5% significance level. These passed the threshold were identified as cue-onset or reward-onset responding cells.

### Sequential activity, neural trajectory analysis, and decision decoder

In Fig. 4, all place-coding cells (defined in Fig. 2) in each trial type were used for sequential activity analysis. For each trial type, the z-scored Ca^2+^ signal from half of the randomized trials was averaged for each cell, and the peak activity sequence was extracted. The averaged Ca^2+^ signals from the other half of the trials were sorted by this sequence. This cross-validation method was used to avoid detecting “false positive” cell activity peak sequences. Then the Ca^2+^ signals from the other trial type were also sorted by the same sequence. For neural trajectory analysis, fluorescence values were interpolated to the same length across trials. PCA was used to reduce dimensionality, and the three principal components that explained the most variance were used to visualize the neural trajectory. SVM classifier (MATLAB application) was used to decode the decision (i.e., trial type) with un-averaged Ca^2+^ traces from all neurons—whether place-coding or non-place-coding—based on the spatial information analysis described above. The model was cross-validated; two-thirds of the trials were used to train the model, and the other one-third were used to test the decoder. The number of trials of each type was balanced. Frames starting from cue onset to the maximum length of all trials were used for visualization in Fig. 5a,c,d.

### Quantification and statistical analysis

All analysis has been estimated with 95% confidence interval and P-values of 0.05 were considered the cutoff for statistical significance. All data are represented as mean ± SEM, and n represents the number of animals unless otherwise specified.

## Supplementary Information

Supplementary Information 1.

Supplementary Video S1.

## Data Availability

All methods are reported in accordance with ARRIVE guidelines.
