# Supplementary material for: Dentate granule cells encode auditory decisions after reinforcement learning in rats
Source: Sci Rep. 2021 Jul 13;11:14360. doi: 10.1038/s41598-021-93721-8 (PMC8277790; doi:10.1038/s41598-021-93721-8)
Supplement: Supplementary file 1 — Supplementary Information 1. [file 41598_2021_93721_MOESM1_ESM.pdf]

Supplementary Figure 1

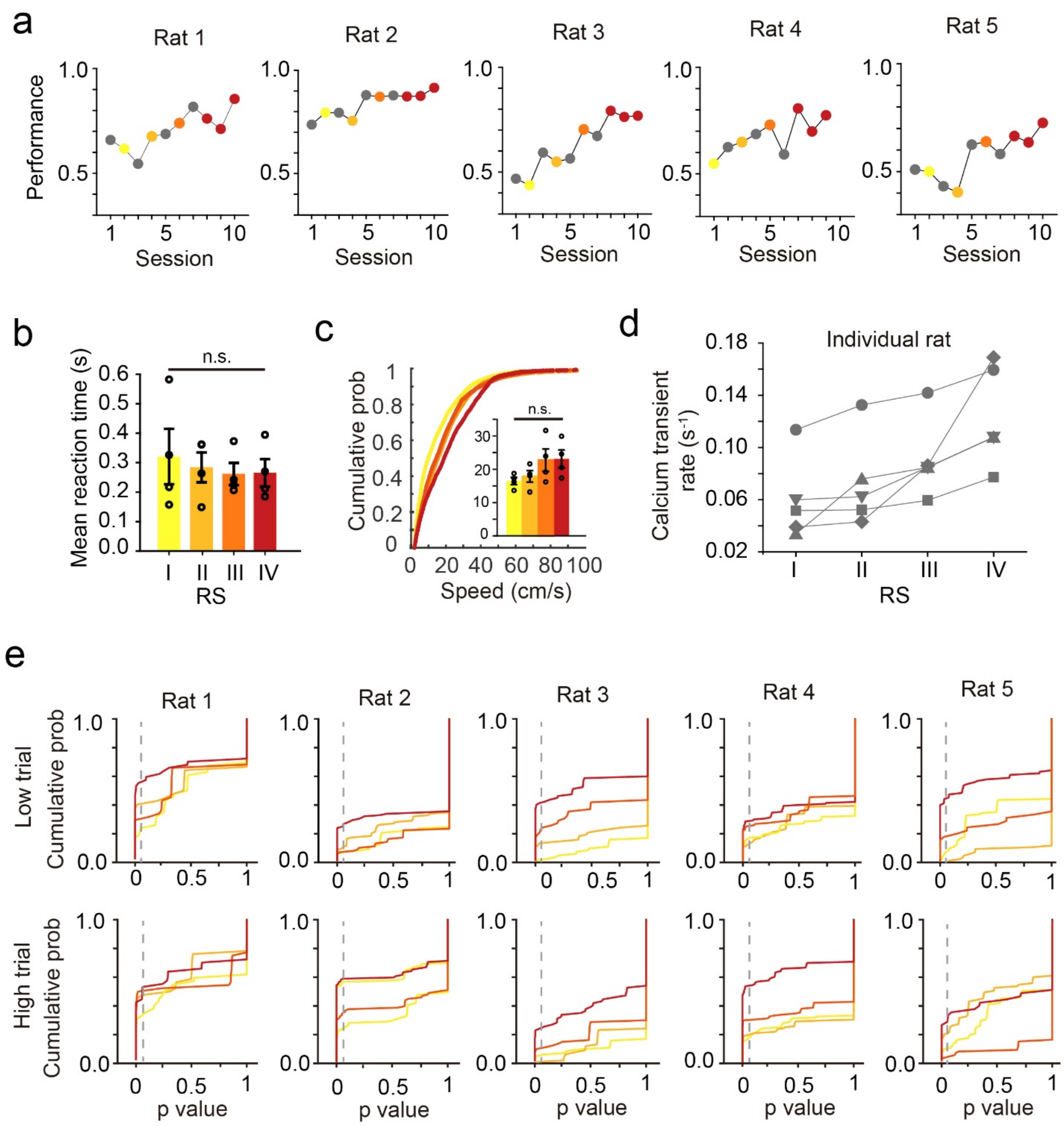

S.Figure1. Task performance, calcium transient rate, and spatial coding in individual rats

a) Task learning progress on task performance of individual rats. The Ca^{2+} imaging sessions were labeled yellow, light orange, dark orange and red. b) Mean reaction time (from cue onset to poke withdrawal) across RSs (n = 4 rats, mean ± SEM, Kruskal-Wallis test, n.s., *P* > 0.05). c)

Cumulative probability of running speed across all learning RSs. d) Mean Ca^{2+} transient rate in each individual rat across four RS. e) Cumulative probability of the *P* values of DGC spatial information in each learning RS for individual rats in low (left) and high (right) trials (dashed line indicates $P = 0.05$).

Supplementary Figure 2

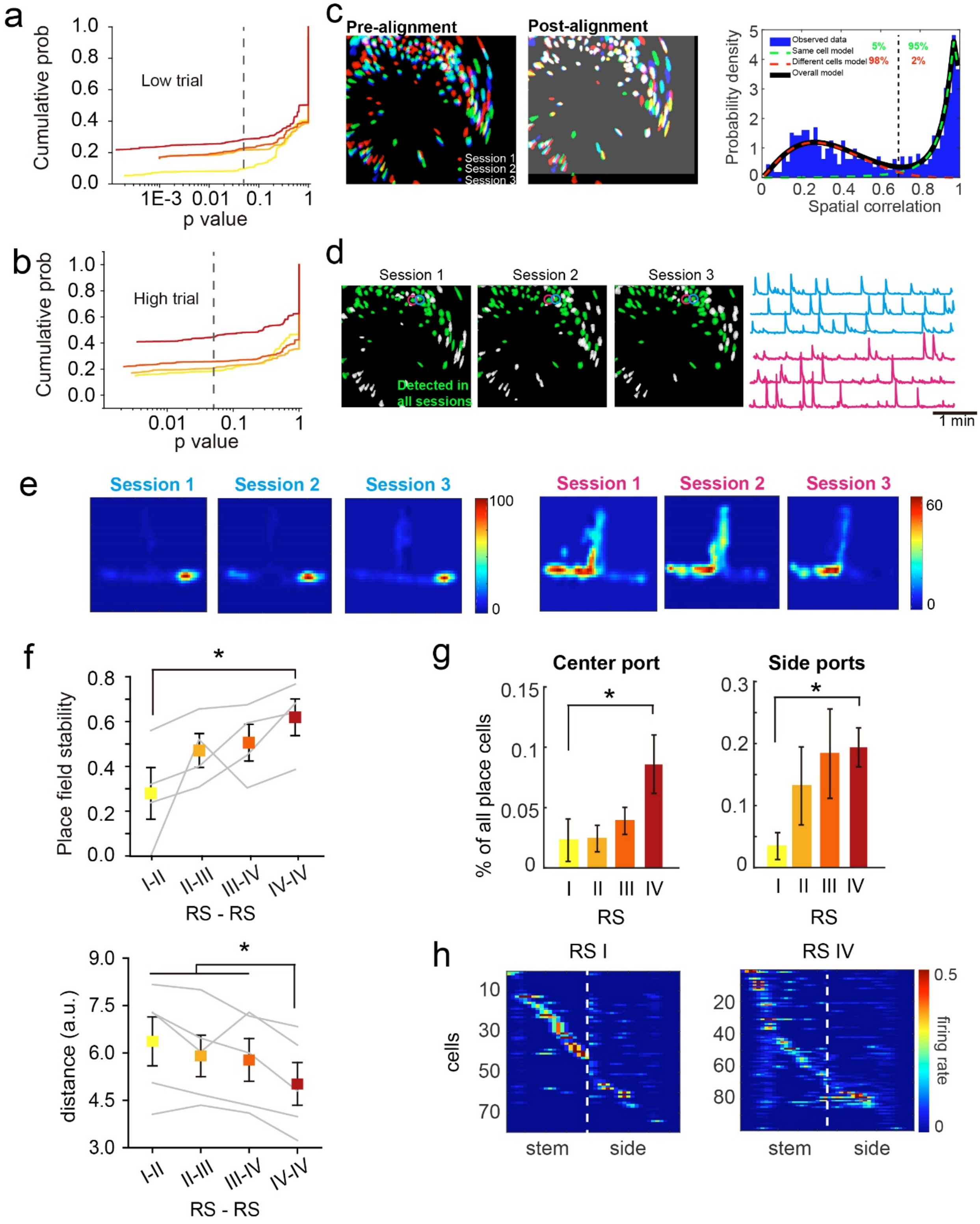

S.Figure 2. Longitudinal cell registration across sessions revealed stable spatial representation after learning

a)-b) Cumulative probability of the *P* values in spatial information analysis in each learning RS and off-task session in low (left) and high (right) trials in log scale (dashed line indicates the place cell threshold *P* value, 0.05).c) All detected cells from the three sessions of RS IV before and after alignment. b) Distribution of spatial correlation in same-cell vs. different-cell models. d) Two adjacent cells detected in all three sessions showing distinct Ca^{2+} signals over time. e) Ca^{2+} intensity maps of these two cells. f) Percentage of cells showing the same place field between two recording sessions and distance between place fields in each RS and different sessions in RS IV. (n = 5 rats, mean ± SEM, Kruskal-Wallis test, *$P < 0.05$). g) Percentage of place cells with highest firing rate at the center or side ports in each stage (n = 5 rats, mean ± SEM, Kruskal-Wallis test, *$P < 0.05$). h) Example of sequentially sorted place cells in RS I and RS IV from one rat to show aggregated center port cells in RS IV.

Supplementary Figure 3

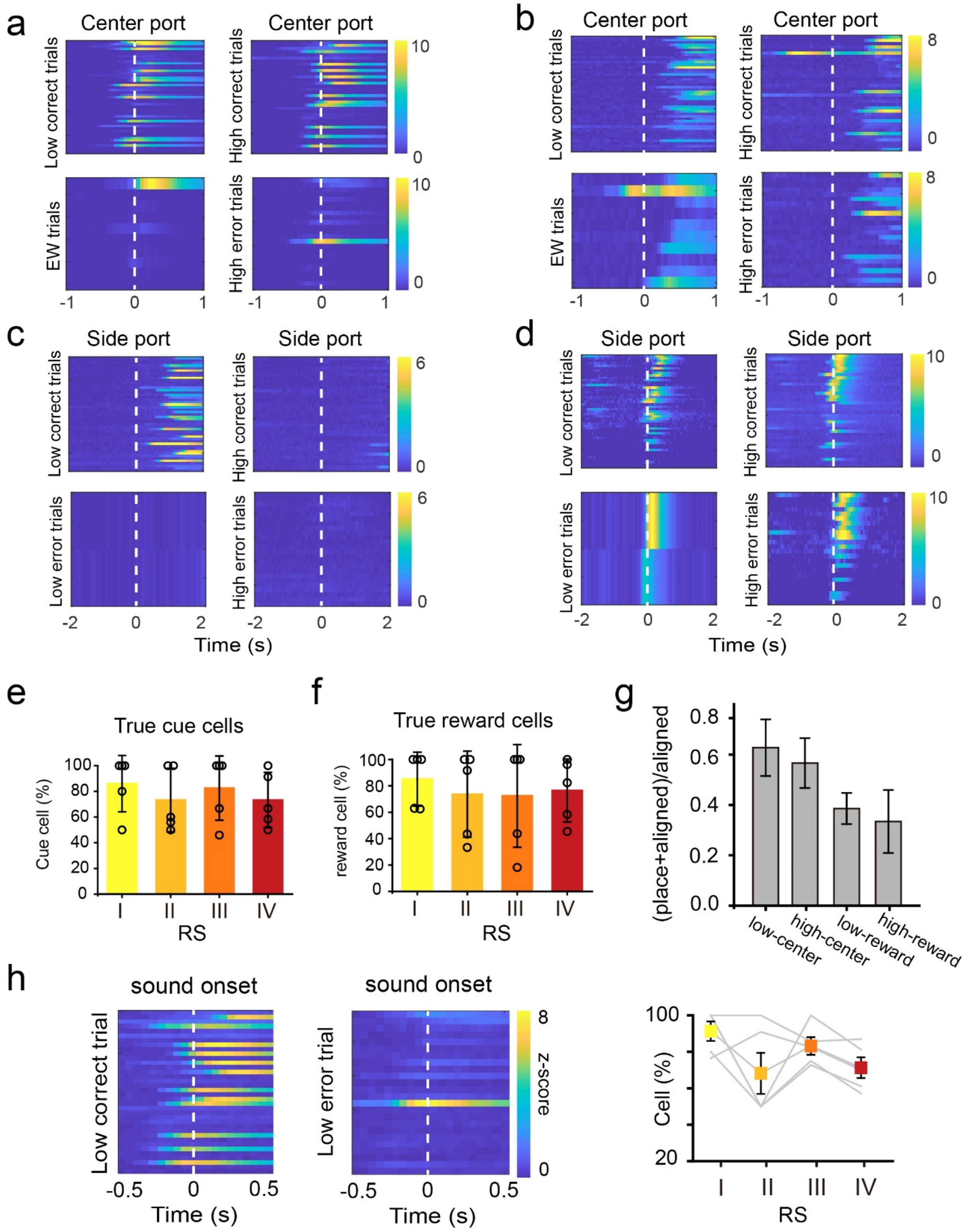

S.Figure 3. DGCs encode task-related events and modulated by learning

a) Example cell responding to successful sound cue triggered trials but not in early withdrawal (EW, no sound triggered) trials. b) Example cell responding to center port poking independent of sound cue. c) Example cell responding to location specific reward receiving but not to reward absence trials. d) Example cell responding to reward port locations (or reward port poking) independent of the presence of reward. e) Percentage of true cue-responding cells (e.g. the cell in a) over the total cells that passed the criteria for cue-onset responding (Fig. 3b and see Methods). h) Percentage of true reward-responding cells (e.g. the cell in c) over the total cells that passed the criteria for activating post reward-onset (Fig. 3d and see Methods). g) Ratio of behavioral-responding cells that were also place-coding. h) Example cell responding to cue onset only in correct trials (left) and the percentage of this type of cell among all cue-onset responding cells (right, n = 5 rats, mean ± SEM, Kruskal-Wallis test, n.s., $P > 0.05$).

Supplementary Figure 4

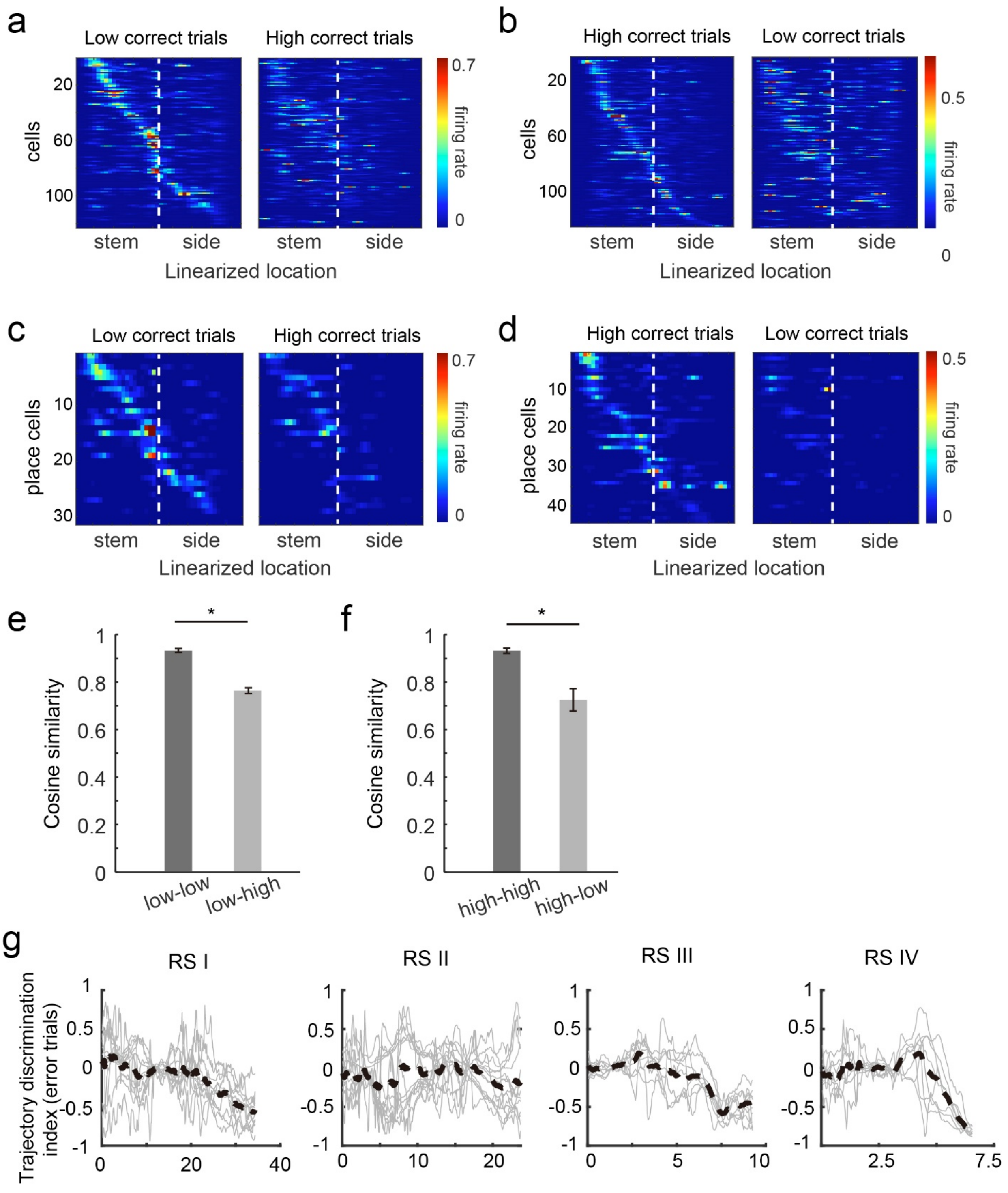

S.Figure 4. **DGC ensembles showed distinct activity patterns to represent spatial decisions**

a) DGC activity (all cells) was sorted by the maximum Ca^{2+} transient rate in low trials over location (spatially binned and linearized locations) and plotted for low trials (left) or high trials (right), white dashed line indicates the turn location in the maze). b) Same as that in b but in a reversed trial type analysis. c) DGC activity (place cells only) was sorted by the maximum Ca^{2+} transient rate in low trials over location and plotted for low trials (left) or high trials (right, white dashed line indicates the turn location in the maze). d) Same as that in c but in a reversed trial type analysis. e) Cosine similarity of Ca^{2+} dynamics from all place cells between two subsets of low trials or between one subset of low trials and high trials (n = 5 rats, Kruskal-Wallis test, mean ± SEM, n.s., $P > 0.05$). f) Same as e but the reversed trial type analysis. g) Neural trajectory discrimination index in error trials.

Supplementary Figure 5

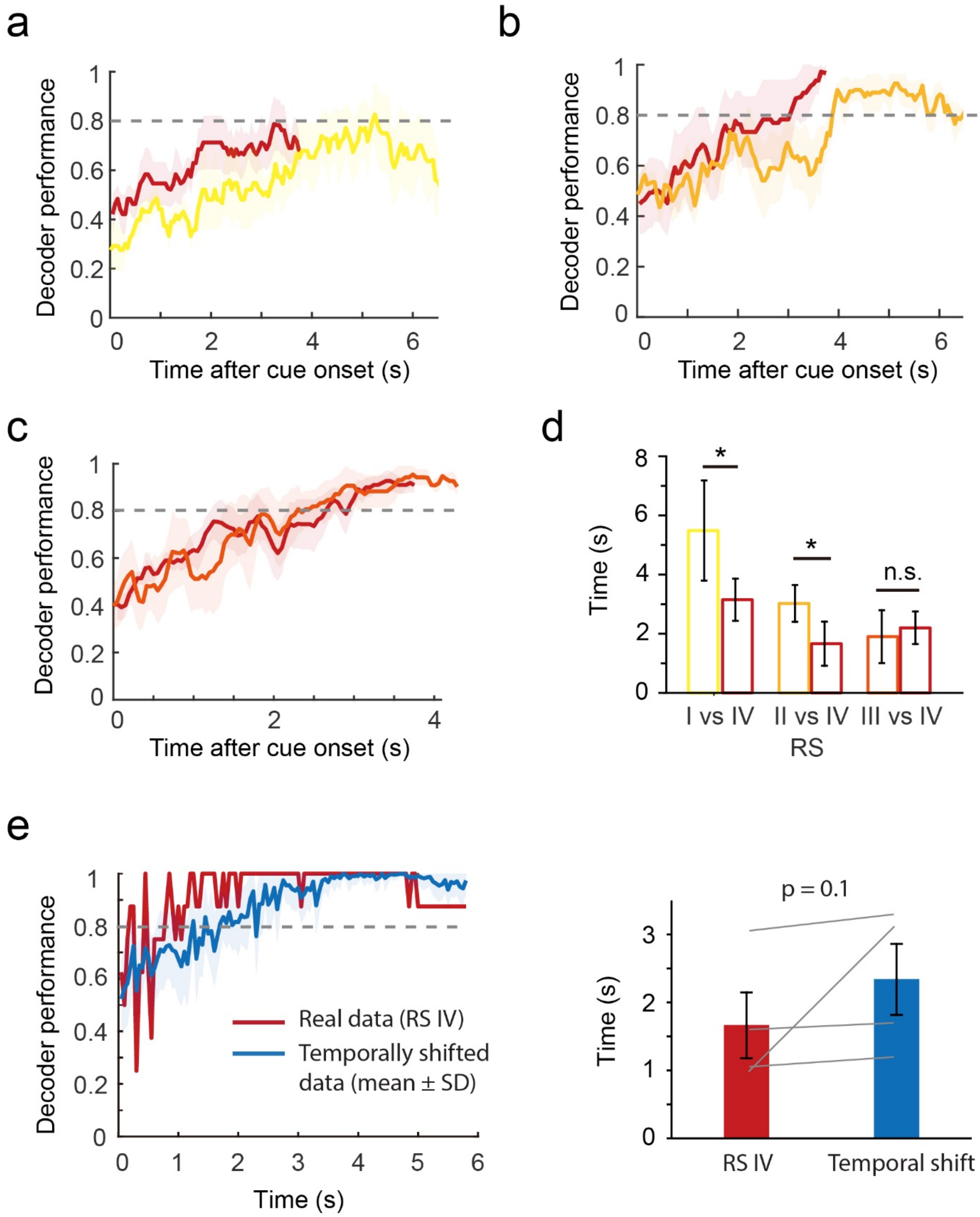

S.Figure 5. Trial number matched decoder performance

a) Decoder performance in RS IV using the number of trials in RS I. b) Decoder performance in RS IV using the number of trials in RS II. c) Decoder performance in RS IV using the number of trials in RS III. d) Average frames needed to reach 80% decoder performance in RS IV using the number of trials in each early RS compared with behavioral performance in early RS (n = 5 rats, mean ± SEM, Wilcoxon signed-rank test, n.s., $P > 0.05$, *$P < 0.05$). e) Left, decoder performance in the RS IV (red, from one example rat) and its temporally shifted data (1000 shuffling, mean ± SD). Right, average frames needed to reach 80% decoder performance in RS IV and temporally shifted data (n = 4 rats, mean ± SEM, Wilcoxon signed-rank test, n.s., $P > 0.05$).

Supplementary Figure 6

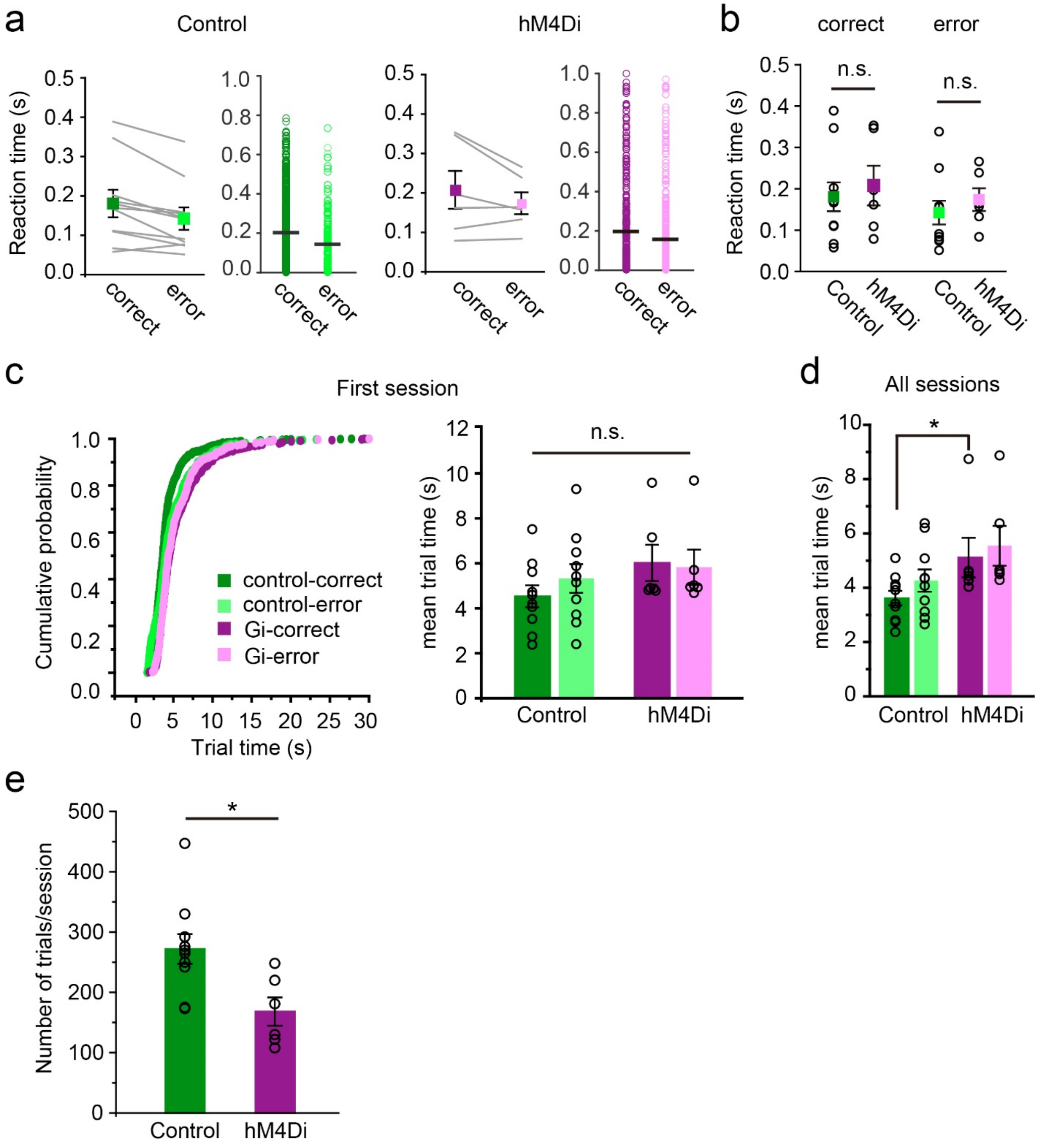

S.Figure 6. Reaction time and mean time spent per trial time in control and hM4Di groups

a) Left, mean reaction times of control rats in sessions with performance <70%with example distributions from one rat. Right, same as left but for rats injected with AAV-hM4Di (n = 10 rats for control, n = 6 rats for hM4Di). b) Comparison of reaction times between control and hM4Di groups

(n = 10 rats for control, n = 6 rats for hM4Di, mean ± SEM, Kruskal-Wallis test, n.s., $P > 0.05$, $^*P < 0.05$).c) Left, distribution of trial times for control and hM4Di-injected rats categorized by correct vs. error trials in the first training session in the Training Phase . Right, average trial time (mean ± SEM, n.s., $P > 0.05$). d) Average trial time for all training sessions in the Training Phase (mean ± SEM, $^*P < 0.05$). e) Average number of trials per session in control and hM4Di group.

Supplementary video

Left, a well-trained rat performing the auditory-cued decision-making task in a three-arm chamber. Right, Δf/f calcium signal of DGCs (850 μm x 650 μm) from the behaving rat. The video plays in 4x fast motion.
